# Substrate specificity of Chondroitinase ABC I based on analyses of biochemical reactions and crystal structures in complex with disaccharides

**DOI:** 10.1093/glycob/cwab086

**Published:** 2021-08-12

**Authors:** Makoto Takashima, Ippei Watanabe, Akimasa Miyanaga, Tadashi Eguchi

**Affiliations:** Department of Chemistry, Tokyo Institute of Technology, 2-12-1 O-okayama, Meguro-ku, Tokyo 152-8551, Japan; Medical Affairs, Seikagaku Corporation, 1-6-1 Marunouchi, Chiyoda-ku, Tokyo 100-0005, Japan; Department of Chemistry, Tokyo Institute of Technology, 2-12-1 O-okayama, Meguro-ku, Tokyo 152-8551, Japan; Department of Chemistry, Tokyo Institute of Technology, 2-12-1 O-okayama, Meguro-ku, Tokyo 152-8551, Japan

**Keywords:** chondroitin sulfate, chondroitinase ABC I, crystal structure, kinetic analysis, substrate specificity

## Abstract

Chondroitinase ABC I (cABC-I) is the enzyme which cleaves the β-1,4 glycosidic linkage of chondroitin sulfate (CS) by β-elimination. To elucidate more accurately the substrate specificity of cABC-I, we evaluated the kinetic parameters of cABC-I and its reactivity with CS isomers displaying less structural heterogeneity as substrates, e.g., approximately 90 percent of disaccharide units in Chondroitin sulfate A (CSA) or Chondroitin sulfate C (CSC) is D-glucuronic acid and 4-*O*-sulfated *N*-acetyl galactosamine (GalNAc) (A-unit) or D-glucuronic acid and 6-*O*-sulfated GalNAc (C-unit), respectively. cABC-I showed the highest reactivity to CSA and CSC among all CS isomers, and the *k*_cat_/*K*_m_ of cABC-I was higher for CSA than for CSC. Next, we determined the crystal structures of cABC-I in complex with CS disaccharides, and analyzed the crystallographic data in combination with molecular docking data. Arg500 interacts with 4-*O*-sulfated and 6-*O*-sulfated GalNAc residues. The distance between Arg500 and the 4-*O*-sulfate group was 0.8 Å shorter than that between Arg500 and the 6-*O*-sulfated group. Moreover, it is likely that the 6-*O*-sulfated group is electrostatically repulsed by the nearby Asp490. Thus, we demonstrated that cABC-I has the highest affinity for the CSA richest in 4-*O*-sulfated GalNAc residues among all CS isomers. Recently, cABC-I was used to treat lumbar disc herniation. The results provide useful information to understand the mechanism of the pharmacological action of cABC-I.

## Introduction

As linear polysaccharides composed of repeating disaccharide units of uronic acid and hexosamine, the glycosaminoglycans (GAGs) are classified into, e.g., chondroitin sulfate (CS), dermatan sulfate (DS), hyaluronan (HA), heparan sulfate (HS) and keratin sulfate (KS). CS is a sulfated polysaccharide composed of repeating of D-glucuronic acid (GlcA) and *N*-acetyl galactosamine (GalNAc) with a β1–3 linkage. It is widely distributed in human or animal tissues and associated with biological and pathological phenomena (Linhardt and Toida 2004; Raman et al. 2005; Gama et al. 2006; Mikami and Kitagawa 2013). CS is classified into six major isomers based on the positions of sulfate groups as follows: chondroitin 4-*O*-sulfate (CSA), chondroitin 6-*O*-sulfate (CSC), chondroitin 2,6-*O*-sulfate (CSD), chondroitin 4,6-*O*-sulfate (CSE) and non-sulfated CS (CH). DS, also known as CSB, is an epimeric form of CSA and contains L-iduronic acid (IdoA) instead of GlcA.

Chondroitin sulfate lyase (CSase) is the enzyme which cleaves the β-1,4 glycosidic linkage of GAG polysaccharides by β-elimination. According to its substrate specificity, CSase is mainly categorized into three types: Chondroitinase ABC (cABC), Chondroitinase AC (cAC) and Chondroitinase B. cABC has been used as a GAG-degrading enzyme for more than 30 years (Murata and Yokoyama 1985a; Yoshida et al. 1989). Two cABC enzymes with different substrate reactivity, Chondroitinase ABC I (cABC-I) and cABC-II, have been identified in *Proteus vulgaris* (Yamagata et al. 1968, Hamai et al. 1997). cABC-I **(**Hernicore®) was developed as a therapeutic agent for lumbar disc herniation (LDH), which has been used in Japan since 2018. The nucleus pulposus (NP) at the center of lumbar disc contains proteoglycan consisting of CS or KS (Olczyk 1993), which forms a gel-like structure with water-retention properties. A bulging NP displaced toward the posterior annulus fibrosus compresses the spinal and nerve roots, causing LDH with various symptoms, e.g., back pain and leg pain. By degradation of CS, cABC-I directly injected into the NP causes reduction in disc pressure and herniation volume, and relieves the symptoms associated with LDH (Sugimura et al. 1996; Takahashi et al. 1996; Yamada et al. 2001).

A 997-amino acid residue monomeric protein, cABC-I, is an endolytic enzyme with broad reactivity to CS isomers as substrates. Although previous studies have shown that cABC-I is highly reactive with CSC, CSA and DS, these CS molecules are derived from animals and contain considerable amounts of various disaccharides that are characteristic of other CS isomers (Suzuki et al. 1968; Volpi 2007). For example, CSC derived from shark cartilage has approximately 70 percent of C-unit [GlcA β1–3 GalNAc (6S)] and 20 percent of A-unit [GlcA β1–3 GalNAc (4S)] and other disaccharide units. Moreover, the molecular weight (Mw) is different for each CS isomer. To evaluate the substrate specificity of cABC-I more accurately, we speculated that it is necessary to use CS molecules with less structural heterogeneity.

Enzyme-substrate complex crystal structure and mutagenesis studies can provide useful information on the function of the amino acid residues in enzymes and their interaction with substrates. However, the crystal structure of cABC-I in complex with its substrates has never been determined. Previous crystallographic and mutagenesis studies of cABC-I alone have demonstrated that the cleft region of this enzyme has six amino acid residues (Arg500, His501, Tyr508, Arg560, His561 Glu653) involved in the reaction with substrates (Prabhakar, Capila et al. 2005a; Prabhakar, Raman et al. 2005b; Kawaguchi et al. 2013). However, the substrate recognition mechanism of cABC-I described above is only a hypothetical model and is not based on insight gained from study of the crystal structure of cABC-I in complex with its substrates. It is thus still unclear which amino acid residues are involved and how each of them works to determine substrate specificity.

In this study, reactivity and kinetic parameters of cABC-I were estimated using CS isomers with less structural heterogeneity as substrates. Further, we succeeded in structural determination of three complexes of cABC-I–CS disaccharide units designated: A-unit, C-unit and non-sulfated unit. In addition, we performed docking analysis of CSA or CSC tetrasaccharide. These data reveal the structural basis of the substrate specificity of cABC-I, which is useful information to understand the mechanism of the pharmacological action of cABC-I in the NP.

## Results

### Reactivity of cABC-I with CS isomers and non-sulfated GAGs

**Fig. 1:**
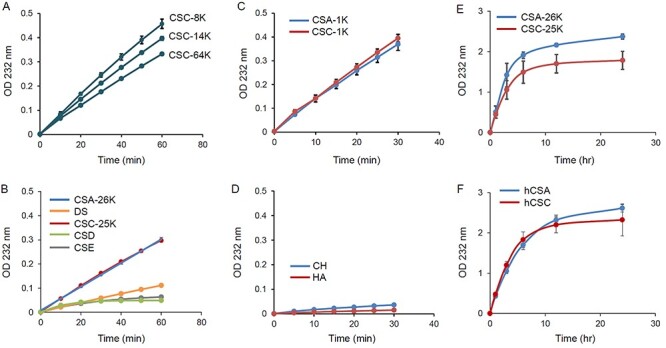
Reactivity of cABC-I with various GAGs. Reactivity of CSCs with various Mws (A), CS isomers with an Mw of 25 kDa (B), CSA and CSC with an Mw of 1 kDa (C), CH and HA (D), enzymatic reaction of CSA and CSC with an Mw of 25 kDa for 24 h (E), enzymatic reaction of hCSA and hCSC for 24 h (F) were determined by time-dependent absorbance at 232 nm of the reaction mixture. The values represent the mean ± standard deviations (*n* = 3).

**Fig. 2:**
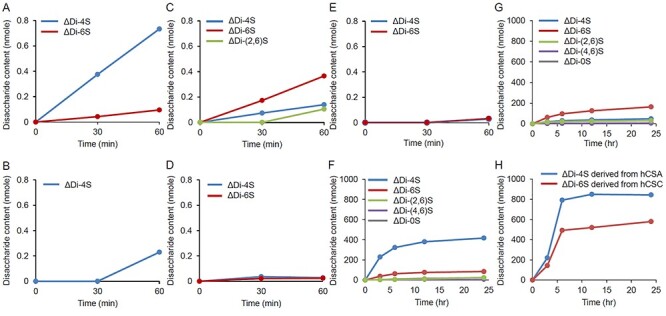
Quantification of generated disaccharides units. The disaccharides produced by enzymatic reaction of CSA, DS, CSC, CSD and CSE with Mw of almost 25 kDa for 60 min (A, B, C, D and E, respectively). The disaccharides produced by enzymatic reaction of CSA and CSC with Mw of almost 25 kDa (F and G, respectively), and hCSA or hCSC for 24 h (H). The disaccharides were determined by HPLC with post column fluorescence detection (*n* = 1).

We used GAGs shown in Table I to evaluate the substrate reactivity of cABC-I. No evidence has yet indicated a relationship between the Mw of CS and the reactivity of cABC-I. To investigate the influence of the Mw of CS on the reactivity of cABC-I, firstly, we used CSCs of Mw: 8 kDa, 14 kDa and 64 kDa. As shown in Figure 1A, the reaction of these CSs with cABC-I proceeded over time. The smaller the Mw of the CSC, the higher was the reactivity. It suggested that in order to investigate substrate reactivity using various CS isomers, the substrates should have the same Mw. Next, we evaluated the reactivity of cABC-I toward five CS isomers adjusted to an Mw of almost 25 kDa, and non-sulfated GAGs as substrates: CSA, DS, CSC, CSD, CSE, CH and HA (Figure 1B). cABC-I had a high reactivity to CSA and CSC compared with other CS isomers, and barely recognized the non-sulfated GAGs CH and HA (Figure 1B and D). Both CS oligosaccharides, CSA and CSC, with an Mw of 1 kDa showed almost the same reactivity as those with an Mw of 25 kDa described above (Figure 1C). The reactivity of DS at 60 min was about one-third of that of CSA and CSC. Although the reactivity of CSD and CSE was similar to that of DS up to 30 min, the reaction then almost stopped. In the quantification of generated disaccharides units of each CS isomer by enzymatic reaction, the D-unit [GlcAβ1–3 GalNAc(2,6-*O*-disulfate)] and E-unit [GlcAβ1–3 GalNAc(4,6-*O*-disulfate)] mostly were not detected (Figure 2).

**Table I:** Mw and disaccharide compositions of GAGs used in enzyme assay

GAG	Mw (kDa)	ΔDi-(%)						Sulfur content (%)
		0S	6S	4S	(2,6)S	(4,6)S	(2,4,6)S	
CSA- 1 K	1.3	1.1	14.3	84.6	0.0	0.0	0.0	6.3
CSA-26 K	26.7	6.3	16.1	77.3	0.1	0.3	0.0	6.1
hCSA	28.1	9.7	0.0	90.3	0.0	0.0	0.0	5.9
DS	24.2	0.3	1.2	98.3	0.1	0.0	0.0	6.4
CSC- 1 K	1.3	1.0	73.7	22.2	0.0	3.0	0.0	6.5
CSC- 8 K	8.9	0.7	76.0	16.5	5.9	0.9	0.0	6.7
CSC-14 K	14.5	0.9	77.2	14.7	6.5	0.6	0.0	6.7
CSC-25 K	25.9	1.6	60.7	23.2	12.1	2.3	0.0	7.0
CSC-64 K	64.1	1.1	74.3	17.2	7.0	0.4	0.0	6.7
hCSC	30.0	9.5	90.5	0.0	0.0	0.0	0.0	5.9
CSD	27.5	0.5	29.8	44.2	23.4	1.9	0.2	7.6
CSE	21.6	10.6	25.6	25.0	0.0	38.8	0.0	7.7
CH	7.3	100	0.0	0.0	0.0	0.0	0.0	0.0
HA	8.3	100	0.0	0.0	0.0	0.0	0.0	0.0

Moreover, we extended the reaction time to 24 h, and evaluated the reactivities of cABC-I to CSA-26 K, CSC-25 K and homogeneous CSA (hCSA) and CSC (hCSC) (Figure 1E and F). The reaction of cABC-I with CSA-26 K or CSC-25 K almost stopped in about 6 h after the start of the reaction, and the OD values of CSA-26 K and CSC-25 K at 24 h was 2.4 and 1.8, respectively. Generated disaccharide of each CS substrate also reached a plateau in about 6 h and the amounts of generated disaccharides derived from CSA tended to be more than that of CSC (Figure 2F and G), which correlated well with the profile of the absorbance of reaction mixture of each CS. Although there was no significant difference between the reactivities of cABC-I to CSA-26 K and CSC-25 K in the first 60 min (Figure 1B), after extending the reaction time to 24 h, it became clear that cABC-I had a higher reactivity with CSA-26 K compared with CSC-25 K. We used hCSA and hCSC as CS molecules with less structural heterogeneity, containing approximately 90 percent ΔDi-4S or ΔDi-6S, respectively (Table I). As shown in Figure 1F, the OD values of hCSA and hCSC at 24 h were 2.6 and 2.3, respectively. The reactivity of cABC-I was dramatically improved for hCSC compared with that for CSC-25 K. For generated disaccharide of each hCS, ΔDi-4S was generated more than ΔDi-6S over the entire measurement point (Figure 2H). The reaction of cABC-I with hCSA began to slow down after 12 h, but unlike CSA-25 K, the OD value continued to rise gradually.

### Kinetic analysis of cABC-I with CSA and CSC

**Fig. 3:**
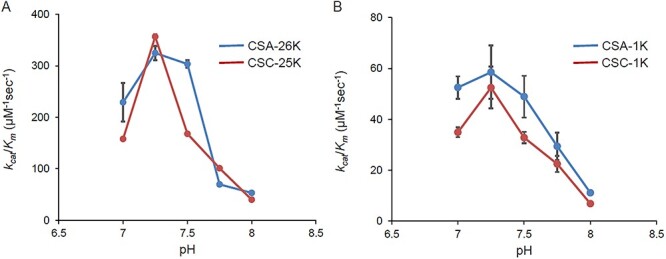
pH-*k*_cat_/*K*_m_ profile for the reaction of cABC-I with CSA and CSC. The *k*_cat_/*K*_m_ for CSA and CSC with Mw of almost 25 kDa at various pH values (A) and that for Mw of 1 kDa (B). The values represent the mean ± standard deviations (*n* = 3).

The *k*_cat_/*K*_m_ for CSA and CSC showed high values in the range of pH 7.0–7.5 (Figure 3A and B). The *k*_cat_/*K*_m_ peak was reached at around pH 7.25, and the values started to decrease when the pH exceeded 7.5, respectively. It suggested that the optimum pH range for cABC-I is from 7 to less than 8, which is close to the body’s own pH environment. As shown in Figure 3, irrespective of Mw, CSA showed higher *k*_cat_/*K*_m_ compared to CSC as follows. The *k*_cat_/*K*_m_ for CSA-26 K was slightly higher than that for CSC-25 K. The *k*_cat_/*K*_m_ for CS oligosaccharides (with Mw of 1 K) also exhibited pH dependence similar to that for CS with Mw of 25 K, and the *k*_cat_/*K*_m_ for CSA-1 K was slightly higher than that for CSC-1 K. The *k*_cat_/*K*_m_ for CS oligosaccharides was approximately one-fifth that for CS-25 K, indicating that the reaction rate constant varied depending on Mw (Figure 3B). As shown in Table II, the *k*_cat_/*K*_m_ for hCSA and hCSC at pH 7.25 was 964 μM^−1^ sec^−1^ and 599 μM^−1^ sec^−1^, respectively. The *k*_cat_/*K*_m_ for each of the hCSs was almost two to three times higher than that for CSA-26 K and CSC-25 K.

**Table II:** Kinetic parameters of cABC-I with hCSA and hCSC

Substrate	*K*_m_ (μM)	*k*_cat_ (sec^−1^)	*k*_cat_ / *K*_m_ (μM^−1^ sec^−1^)
hCSA	1.56 ± 0.01	1510 ± 130	964 ± 74
hCSC	2.11 ± 0.54	1210 ± 83	599 ± 153

The values represent the mean ± standard deviations (*n* = 3).

### Overall structures of cABC-I complexes with disaccharides

**Fig. 4:**
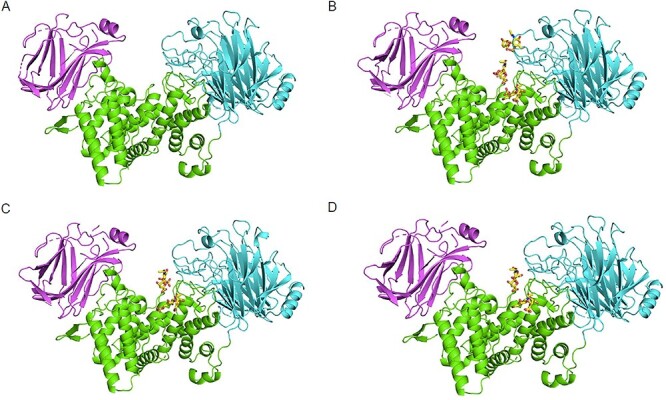
Overall structure of cABC-I. The crystal structure of cABC-I (PDB ID: 7EIP), complex with ΔDi-4S (PDB ID: 7EIQ), ΔDi-6S (PDB ID: 7EIR) and ΔDi-0S (PDB ID: 7EIS) are shown as a cartoon model (A, B, C and D, respectively). Colors: N-terminal domain (25–234) – magenta, middle domain (235–617) – green, C-terminal domain (618–1021) – cyan; the disaccharides are shown as yellow sticks.

The structures of ligand-free cABC-I and cABC-I in complex with disaccharides (ΔDi-4S, ΔDi-6S or ΔDi-0S), the final product of the enzymatic reaction, have been determined at 1.88 Å, 1.80 Å, 1.92 Å and 2.50 Å resolutions, respectively (Figure 4 and Supplementary Table SI). These disaccharides were bound to cABC-I at the active site cleft, specifically at the amino acid residues including Arg500, His501, Tyr508 and Arg560. The overall structures of cABC-I bound to these disaccharides were almost identical to those of the unbound form (Cα r.m.s.d.: cABC-I-ΔDi-4S complex, 0.131 Å; cABC-I-ΔDi-6S complex, 0.161 Å; cABC-I-ΔDi-0S complex, 0.217 Å), indicating that cABC-I was not conformationally changed by interaction with disaccharides. According to the electron density analysis, the number of disaccharides bound to a single molecule of cABC-I was as follows: ΔDi-4S, 3 molecules; ΔDi-6S, 2 molecules; ΔDi-0S, 2 molecules (Supplementary Figure SI).

### Interaction of cABC-I with ΔDi-4S

**Fig. 5:**
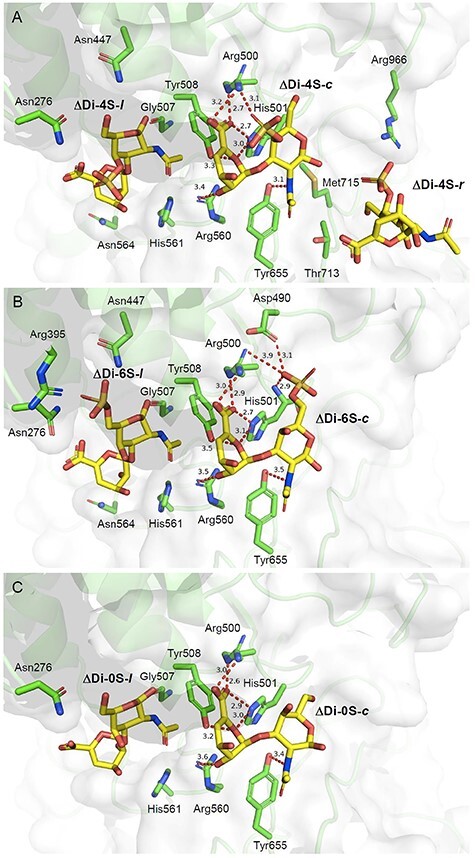
Interaction of cABC-I with ΔDi-4S, ΔDi-6S and ΔDi-0S (A, B and C, respectively). The disaccharides and the residues involved in interactions are shown as yellow and green sticks, respectively. Key interactions are shown in red dashed lines.

Three molecules of ΔDi-4S were bound to the active site cleft of cABC-I, as shown in Figure 5A. The disaccharide bound to the center of the active site cleft is labeled as ΔDi-4S-*c*, and the disaccharides located to the left and right of ΔDi-4S-*c* are labeled as ΔDi-4S -*l* and -*r*, respectively. The 4-*O*-sulfate group in GalNAc of ΔDi-4S-*c* forms a salt bridge (3.1 Å) with the side chain of Arg500. The interactions between each functional group of ΔGlcA and the amino acid residues of cABC-I involved in the reaction are as follows: the O5 oxygen interacts with the side chain of His501 and Tyr508; the carboxy group interacts with the side chain of His501 and Arg500; the 2-hydroxyl group interacts with the side chain of Arg560. The interactions between the functional groups of ΔDi-4S (−*r* or −*l*), and amino acid residues are summarized in Table III.

**Table III:** Distance between the functional groups of disaccharides and interacting amino acid residues

Region	Sugar		Protein		Distance (Å)		
	Residue	Atom	Amino acid	Atom	ΔDi-0S	ΔDi-4S	ΔDi-6S
-*l*	ΔGlcA	O2	Asn564	ND2	-	3.1	3.4
	GalNAc (sulfate)	O1	Gly507	O	-	3.3	3.2
		N			3.1	3.1	3.5
		O6	Asn276	OD1	3.1	3.1	-
				ND2	-	-	3.4
			Asn447	ND2	-	3.4	-
		6O2S	Asn276	ND2	-	-	3.3
			Arg395	NH1	-	-	3.5
			Asn447	ND2	-	-	3.6
		6O3S	Asn276	ND2	-	-	2.3
		O7	Arg560	NH1	3.2	3.4	3.1
			His561	NE2	3.1	2.8	2.8
-*c*	ΔGlcA	O2	Arg560	NH1	3.6	3.4	3.5
		O5	His501	NE2	3.0	3.0	3.1
			Tyr508	OH	3.2	3.3	3.5
		O6A	Arg500	NH2	3.0	3.2	3.0
		O6B	Arg500	NH2	2.8	2.9	3.0
				NE	2.6	2.7	2.9
			His501	NE2	2.9	2.7	2.7
	GalNAc (sulfate)	N	Tyr655	OH	3.4	3.1	3.5
		4O3S	Arg500	NH2	-	3.1	-
		6O2S	Arg500	NH1	-	-	3.9
			His501	N	-	-	2.9
-*r*	ΔGlcA	O6A	Thr713	N	-	3.0	-
		O6B		OG1	-	2.8	-
	GalNAc (sulfate)	4O2S	Arg966	NH2	-	3.0	-
		O6	Met715	N	-	2.8	-

### Interaction of cABC-I with ΔDi-6S

Two molecules of ΔDi-6S (−*c* or −*l*) were bound to the active site cleft of cABC-I, as shown in Figure 5B, and the binding position of each disaccharide was similar to that of ΔDi-4S (−*c* or −*l*). The 6-*O*-sulfate group in GalNAc of ΔDi-6S-*c* forms a salt bridge (3.9 Å) with the side chain of Arg500. The length of the salt bridge with the 6-*O*-sulfate group is 0.8 Å longer than that with the 4-*O*-sulfate group. In addition, the 6-*O*-sulfate group also interacts with the main chain of His501. However, the 6-*O*-sulfate group is located close to the side chain of Asp490 (3.1 Å), suggesting the existence of electrostatic repulsion. The interactions between each functional group of ΔGlcA and the amino acid residues are almost identical to that of ΔDi-4S-*c*. The interactions between the functional groups of ΔDi-6S-*l* and amino acid residues are summarized in Table III.

### Interaction of cABC-I with ΔDi-0S

Two molecules of ΔDi-0S (−*c*, −*l*) were bound to the active site cleft of cABC-I, as shown in Figure 5C, and the binding position of each disaccharide was similar to that of ΔDi-4S (−*c* and −*l*). The GalNAc moiety in ΔDi-0S-*c* does not interact with any amino acid residues, except for hydrogen bond between the amide nitrogen and the side chain of Tyr655. The interactions between each functional group of ΔGlcA and the amino acid residues are almost identical to those of ΔDi-4S-*c* and ΔDi-6S-*c*. The interactions between the functional groups of ΔDi-0S-*l* and amino acid residues are summarized in Table III.

### Docking analysis

**Fig. 6:**
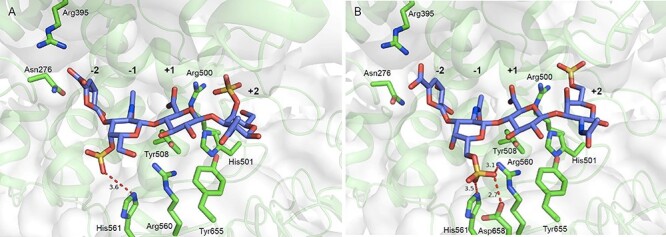
Structure of cABC-I active site in the docking model with CSA and CSC tetrasaccharides (A and B, respectively). Each tetrasaccharide and residues involved in interactions are shown as slate blue and green sticks, respectively. Key interactions are shown in red dashed lines.

The result of docking analysis of CSA tetrasaccharide (subsites −2, −1, +1 and + 2) is shown in Figure 6A. The position of the GalNAc moiety of ΔDi-4S-*c* in the complex structure was well superimposed on subsite +2, suggesting that the binding positions of GalNAc in both analyses were almost identical. These results suggested that the binding positions of GalNAc in subsite −1 could be described more precisely based on the data obtained in the docking study. However, the position of the GlcA moiety (subsite +1) differed by approximately 30° compared with that of ΔGlcA of ΔDi-4S-*c* (Supplementary Figure SIIA). This difference is probably due to the fact that ΔGlcA of ΔDi-4S-*c* has a double bond. The position of GalNAc and ΔGlcA moieties of ΔDi-4S-*l* were greatly deviated from subsites −1 and − 2, respectively, based on the comparison of the binding positions between the crystal structure and docking model. Docking analysis showed that the 4-*O*-sulfate group of GalNAc (subsite −1) interacts with the side chain of His561.

The result of docking analysis of CSC tetrasaccharide (subsites −2, −1, +1 and + 2) is shown in Figure 6B. As in the result of docking analysis using CSA tetrasaccharide described above, the binding positions of GalNAc in ΔDi-6S-*c* and subsite +2 well agreed with each other, but the position of GlcA moiety (subsite +1) was slightly different compared with the position of ΔGlcA of ΔDi-4S-*c*. The position of the GalNAc and ΔGlcA moieties of ΔDi-6S-*l* were greatly deviated from subsites −1 and − 2, respectively, in combined crystal structure and molecular docking analyses (Supplementary Figure SIIB). Docking analysis showed that the 6-*O*-sulfate group of GalNAc (subsite −1) interacts with the side chain of Arg560 and His561; however, the distance to Asp658 was short enough (2.7 Å) to possibly cause electrostatic interaction.

## Discussion

Many papers have reported the substrate reactivity of cABC-I, particularly its high reactivity with CSA and CSC (Hamai et al. 1997; Prabhakar, Raman et al. 2005b). Various CS isomers derived from animals have a wide range of Mw and the disaccharide composition of individual CS molecules can vary. We hypothesized that differences in CS Mw and disaccharide composition affect the reactivity of cABC-I even when the CS is the same isomer. That is, we thought that in order to evaluate the substrate specificity of cABC-I more accurately, we needed to use CSs that are more homogeneous in structure. When CS is cleaved by cABC-I, an unsaturated bond is generated between the C4 and C5 positions of uronic acid at the nonreducing terminus and shows an ultraviolet absorption maximum of around 230 nm. Following previous test methods (Prabhakar, Raman et al. 2005b), we evaluated the reactivity and enzyme kinetics of cABC-I with various CS isomers using a method based on measurement of absorbance at 232 nm.

The reactivity of cABC-I with CS isomer adjusted to an Mw of almost 25 kDa was as follows: CSA > CSC > DS > CSE ≈ CSD (Figure 1B and E). As shown in Table I, CSC contains 14 percent disulfated disaccharides. We speculated that cABC-I is unlikely to react with disulfated disaccharides because the reaction of cABC-I with CSE and CSD reached a plateau in about 30 min, and no Di-(4,6)S and Di-(2,6)S disulfated disaccharides contained in CSE or CSD were detected (Figure 1B, Figure 2D and E). To remove the influence of disulfated disaccharides, we evaluated the reactivity with hCSA and hCSC as CS molecules with less structural heterogeneity, containing approximately 90 percent of ΔDi-4S or ΔDi-6S, respectively. Considering the reactivity of cABC-I with CS isomers as shown in Figures 1 and 2, CSA containing A-unit were the most reactive with cABC-I.

However, reactivity alone is not sufficient to investigate enzyme-substrate affinity. Generally, the kinetic parameter *k*_cat_/*K*_m_ is used as an indicator of enzyme-substrate specificity (Sárkány et al. 2001). In order to evaluate detailed substrate specificity of cABC-I, we created pH- *k*_cat_/*K*_m_ profiles for CSA and CSC by Mw (Figure 3). In general, reactivity between a protein and a polysaccharide depends on the Mw of the polysaccharide: the higher the Mw is, the higher the affinity with the protein is (Watanabe et al. 2015). Therefore, we presumed that also in this study, the higher the Mw of CS was, the higher the affinity of cABC-I for the CS substrate was (Figure 3). Meanwhile, the reactivity increased with decreasing Mw of CS (Figure 1A), and this suggests higher *k*_cat_ values. In fact, the *k*_cat_ of the CS-1 kDa was found to be higher than that of the CS-25 kDa. The amount (molar number) of enzymatic degradation product per unit time reportedly increased with decreasing Mw of substrate in polysaccharide enzymolysis (Zhang and Neau 2001). The cABC-I reaction speed (Vmax) was also reported to increase with the decreasing Mw of CS, suggesting higher *k*_cat_ values. Judging from these facts, CS isomers of lower Mw were considered to have higher reaction speeds and lower enzyme affinity than those of higher Mw. The kinetic parameters of cABC-I should be linked to properties of CS, e.g., Mw and disaccharide composition. Based on these findings, we evaluated the kinetic parameters at pH 7.25 for hCSs with an adjusted Mw below 30 kDa. As shown in Table II, the *k*_cat_/*K*_m_ for hCSA was almost 1.6 times higher than that for hCSC. It indicated that the specificity of cABC-I is the highest for A-unit, which is abundant in CSA. Moreover, the *k*_cat_/*K*_m_ for each hCS was almost two to three times higher than that for CSs with greater structural heterogeneity. Structural heterogeneity in CS molecules such as those with disulfated disaccharides may be inferred from the reactivity of cABC-I with A-unit or C-unit.

Structural analysis of complex with substrates is a useful method to understand the details of enzyme-substrate specificity. Although the crystal structure of ligand-free cABC-I has been determined in previous study (Huang et al. 2003), but the crystal structure of cABC-I in complex with substrate or with CS disaccharide has not been reported. In general, it is difficult to obtain complex with bound substrates such as polysaccharides once enzymatic degradation of the substrate has taken place. In fact, we attempted to soak cABC-I crystals with CS oligosaccharides, but the determined cABC-I structure contained disaccharides, the final product of the enzymatic reaction, in the active site cleft (data not shown). We have successfully determined the crystal structure of cABC-I in complex with the CS disaccharides, ΔDi-4S, ΔDi-6S and ΔDi-0S by soaking cABC-I crystals with these CS disaccharides. As shown in Figure 5, two or three molecules of each disaccharide were bound to cABC-I at the cleft region, where the amino acid residues (Arg500, His501, Tyr508, His561 and Arg560) exist. A previous analysis of the crystal structure of hyaluronidase in complex with ΔDi-HA indicated that two disaccharide molecules are bound to the cleft region at the “substrate recognition area” and “product release area”, respectively (Ponnuraj and Jedrzejas 2000). Similarly, in cABC-I, ΔDi-X-*c* may bind to the former and ΔDi-X-*l* to the latter, playing different roles.

The CS disaccharide used for crystal structural analysis has ΔGlcA with a double bond, which is not present in the undigested polysaccharide CS. As reported in the crystal structure of hyaluronidase in complex with ΔDi-HA (Ponnuraj and Jedrzejas 2000), the presence or absence of the double bond in ΔGlcA may cause a slight shift in the position of the interaction between the functional groups and the amino acid residues in CS. Meanwhile, the complex with the final product does not mimic the binding of the enzyme to the substrate during the reaction. In order to identify the binding site more precisely, we performed a docking analysis of CSA and CSC tetrasaccharides, both of which do not have the double bonds, and compared the results with those from the complex structure analysis using the unsaturated disaccharide. Previous studies have demonstrated that mutation of the following amino acid residues: Arg500, His501, Tyr508 and Arg560, significantly reduces or abolishes the activity of cABC-I (Supplementary Table SII). Considering (i), (ii) and (iii) below, the result of the present docking analysis was consistent with that of the previous mutagenesis study, suggesting the usefulness of the analytical method in this study; (i) the distance between the carboxy group of GlcA (subsite +1) and Arg500 is approximately 3 Å, (ii) the distance between the C5 of GlcA (subsite +1) and His501 is approximately 3 Å, (iii) the distance between the oxygen of the glycosidic bond and Tyr508 and Arg560 is 3 to 4 Å. The docking study made it possible to analyze the interactions between sulfate groups of GalNAc (subsite −1) and amino acid residues.

In this study, we demonstrated for the first time that Arg500 interacts with the 4-*O*- and 6-*O*-sulfate groups of GalNAc bound to the center of the active site cleft (Figure 5). The result of docking analysis also suggested that the side chain of His561 interacts with the 4-*O*-sulfate group, and the side chains of Arg560 and His561 interact with the 6-*O*-sulfate group (subsite −1, Figure 6). The amino acid residues of cABC-I presumably recognize GalNAc in a different manner depending on whether the sulfate groups of GalNAc are in position 4 or 6. Furthermore, the affinity of cABC-I for 4-*O*-sulfate groups was suggested to be higher than that for the 6-*O*-sulfate groups. The basis for this inference is that: (i) the distance between Arg500 and each sulfate group in GalNAc was 0.8 Å shorter for the 4-*O*-sulfate group than for the 6-*O*-sulfate group (Figure 5), and (ii) the 6-*O*-sulfate group of GalNAc (subsite +2 or −1) was close to acidic amino acid residues such as Asp490 and Asp658, which may result in electrostatic repulsion (Figure 6). This electrostatic repulsion may contribute to keeping Arg500 and the 6-*O*-sulfate groups away from each other. Our results thus suggested that cABC-I has the highest affinity for the A-unit-rich CSA molecule among the CS isomers.

**Fig. 7:**
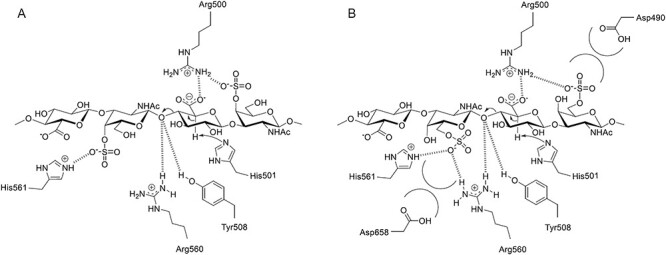
Schematic illustration of the reaction mechanism of cABC-I. (a) CSA substrate in the active site of cABC-I. (b) CSC substrate in the active site of cABC-I.

The roles of these amino acid residues based on the mutagenesis studies are proposed to be as follows: (i) His561 interacts with the sulfate group, (ii) Arg500 plays a role in the charge neutralization of the carboxy group, (iii) His501 abstracts the proton from C5 and (iv) Tyr508 and Arg560 donate a proton to the oxygen of the glycosidic bond (Supplementary Table SII). Hence, the catalytic mechanisms were estimated on the basis of a comprehensive evaluation of published data, and our results for the demonstrated positional relationships between amino acids and substrate functional groups are shown in Figure 7. Arg500 and His561 mutations were reported to reduce the substrate reactivity of cABC-I but, unlike other amino acid mutations, they did not completely deactivate cABC-I (Supplementary Table SII). Hence, the two amino acid residues were suggested to be important to the recognition of the sulfate group of GalNAc by CS, and not to be involved directly in the cleavage of the CS chain, like other amino acid residues involved in the reaction. The reaction between cABC-I and the substrate was suggested to begin with the interaction of the sulfate group of CS with Arg500 and His561, and to proceed to the above-described reactions (iii) and (iv).

The information on the substrate specificity of cABC-I obtained from this study may be useful in understanding the mode of action of the enzyme, but this study has several limitations. The reactivity for the substrate of cABC-I in in vitro experiments does not completely reproduce real activity in the NP because of differences between the laboratory environment and the actual bio-environment (Figures 1 and 2). To assess quantitatively the pharmacodynamics of cABC-I in the living body, ex vivo testing by using NP is needed. CS molecules had structures that were resistant to cABC-I attack according to Sugahara et al. (1994). Such structures may have affected the reactivity of cABC-I with CS, and resulted in stopping reaction as shown in Figure 1E and F. Evaluating the reactivity of cABC-I to CS, including the enzyme’s resistant structures in the NP, would provide information on the real activity of cABC-I in the living body. Further studies are needed to elucidate the detailed the mode of action of the enzyme.

This study is the first to report the substrate specificity of cABC-I, based on the reactivity with CS of low structural heterogeneity and the structural analysis of complex with disaccharides. cABC-I is used as a therapeutic agent for LDH. We mainly used CSA and CSC to evaluate the substrate specificity of this enzyme, because A- and C-units are the dominant forms of GalNAc residues in the NP (Olczyk 1993). The in vitro test results from this study would serve as a basis for understanding the pharmacokinetics of cABC-I in the living body.

## Materials and methods

### Glycosaminoglycans

GAGs used in this study are summarized in Table I. CSA from a whale cartilage was purchased from Carbosynth Ltd. (Berks, UK). DS from a chicken’s cockscomb, CSC from shark cartilage, CSD from shark cartilage, CSE from squid cartilage, HA from a chicken’s cockscomb and CH from recombinant *Escherichia coli* were obtained from Seikagaku Corp. (Tokyo, Japan). Homogeneous CSA (hCSA) and CSC (hCSC) prepared from CH according to the method of Sugiura et al. (2011). GAGs, except for CSA-26 K and DS, were hydrolyzed with hydrochloric acid to reduce the Mw below 30 kDa. The average Mw and disaccharide composition of GAGs were estimated by gel exclusion column chromatography according to the method of Watanabe et al. (2015). The average Mw of CSA-1 K and CSC-1 K were estimated by LC–MS. The LC–MS system used for this study consisted of an LC-20 AD Prominence liquid chromatograph connected to a LC–MS-IT-TOF (Shimadzu Corporation, Japan). Instrument control, data acquisition and processing were accomplished using Shimadzu LCMS solution version 3.80. Briefly, the analysis was performed on a YMC Diol-300 column (8.0 mm × 500 mm) with 10 mM ammonium formate/MeOH (90:10, v/v) as a mobile phase at a flow rate of 0.5 mL/min at 25°C, and in negative ion mode polarity. The MS recording range was 200–1200 in MS mode and 75–2000 in MS/MS mode. Other MS parameters are shown below: detector voltage; 1.5 kV, CDL; 300°C, heat block; 200°C, nebulizer gas; 1.5 L/min.

### Unsaturated disaccharides for crystal structural analysis

Unsaturated disaccharides, ΔDi-4S, ΔDi-6S and ΔDi-0S were prepared according to the method of Murata and Yokoyama (1985b, 1986). Each disaccharide composition was more than 99 percent.

### Enzyme purification

Extraction and purification of cABC-I was performed according to the method of Hamai *et al* (1997). Briefly, *P. vulgaris* NCTC 4636 was cultured at 30°C in the medium containing peptone, meat extract, yeast extract and NaCl with CSC as the inducer. The cells were harvested by centrifugation at the end of logarithmic phase. Then cABC-I was extracted using polyoxyethylene lauryl ether and then purified by ion-exchange resin.

### Reactivity of cABC-I with CS isomers and non-sulfated GAGs

Reactivity of cABC-I was measured by absorbance at 232 nm as described by Yamagata et al. (1968). Exactly 1.0 mL of 0.05 percent substrate in 50 mM Tris–HCl Buffer was mixed with 2.0 mU of cABC-I and incubated for 60 min or for 24 h at 37°C (*n* = 3). Time-dependent absorbance at 232 nm of the mixture was measured using a spectrophotometer (UV-1900i, Shimadzu Corporation). Moreover, each disaccharide generated (μg/mL) by the enzymatic reaction within 60 min was quantified according to the method of Watanabe et al. (2015).

### Kinetic analysis of cABC-I with CSA and CSC

The kinetic parameters of cABC-I reacting with CSA-26 K, CSC-25 K, CSA-1 K and CSC-1 K were calculated based on the Michaelis–Menten equation. To investigate the pH dependence of the kinetic parameters, each substrate was dissolved in 50 mM Tris–HCl Buffer adjusted to pH 7.0, 7.25, 7.5, 7.75 and 8.0, respectively (*n* = 3). The kinetic parameters of cABC-I reacting with hCSA and hCSC at pH 7.25 were calculated by the same procedure. The amount of CS (μM) in each solution was measured by a carbazole-sulfuric acid method (Dische 1947). Protein concentrations were determined by the Lowry method (Lowry et al. 1951) using bovine serum albumin as the standard. cABC-I was mixed with each substrate solution, and the absorbance at 232 nm (P) was measured several times (t) at 37°C. The value of the initial velocity (*v_0_*) was calculated by using the following equation [1].

*v_0_* = d[P]/dt = (P_1_–P_0_)/(t_1_–t_0_)(1)

The 1/*v_0_* was plotted versus the reciprocal of substrate concentration (1/[S]) for five different concentrations and the data were fitted to a straight line. The maximum rate (*V*_max_) and Michaelis–Menten constant (*K*_m_) were extracted from the slope and y-intercept of the Lineweaver–Burk plot generated by plotting 1/*v_0_* and 1/[S] according to equation [2].

[S]/*v_0_* = (1/*V*_max_) [S] + (*K*_m_/*V*_max_)(2)

Turnover number (*k*_cat_) was calculated by dividing *V*_max_ by the concentration of enzyme [E]_0_.

*k*_cat_ = *V*_max_/[E]_0_(3)

### Crystallization, data collection and structural determination

Crystals of cABC-I were grown from a 1:1 mixture of a protein solution (7.5 mg/mL in 20 mM Tris·HCl [pH 7.4]) and a reservoir solution (200 mM magnesium acetate, 200 mM ammonium acetate, 17.5 percent [vol/vol] polyethylene glycol 3350 and 10 mM HEPES-Na [pH 7.5]) by using the sitting-drop vapor-diffusion method at 20°C. Crystals were then soaked in cryoprotectant solution (200 mM magnesium acetate, 200 mM ammonium acetate, 30 percent [vol/vol] polyethylene glycol 3350 and 10 mM Tris–HCl [pH 7.5]) and flash-frozen in a liquid nitrogen stream. Crystals of cABC-I in complex with ΔDi-4S, ΔDi-6S or ΔDi-0S were prepared by soaking ligand-free cABC-I crystals in cryoprotectant solution containing 100 mM of these disaccharide for 30–90 min and were then flash-frozen in a liquid nitrogen stream. X-ray data were collected by using beamlines AR-NW12A and BL5A (Photon Factory, Tsukuba, Japan). All diffraction data were indexed, integrated and scaled by using the program XDS (Kabsch 2010). The initial phases were determined by the molecular replacement method using the program MOLREP (Vagin and Teplyakov 2010), with the previously reported structure of cABC-I (PDB ID 1HN0; Huang et al. 2003) used as a search model. Coot (Emsley and Cowtan 2004) was used for visual inspection and manual rebuilding of the model. Refmac (Murshudov et al. 1997) was used for refinement. The figures were prepared by using PyMOL. Data collection and processing statistics are summarized in supplemental material (Supplementary Table SI). The atomic coordinates and structure factors have been deposited in the Protein Data Bank: PDB entry 7EIP, ligand-free cABC-I; 7EIQ, cABC-I in complex with ΔDi-4S; 7EIR, cABC-I in complex with ΔDi-6S; 7EIS, cABC-I in complex with ΔDi-0S.

### Docking analysis

The docking study was carried out by using the AutoDock program (Version 4.2; Morris et al. 2009). The CSA tetrasaccharide and CSC tetrasaccharide molecules were generated by using the PRODRG2 Server (Schüttelkopf and van Aalten 2004). The crystal structure of ligand-free cABC-I was used for the docking study. All of the water molecules were removed for the docking study. By using AutoDockTools, polar hydrogen atoms were added to amino acid residues, and Gasteiger charges were assigned to all atoms of the protein. The sugar rings of the ligand molecule were fixed in the ^4^C_1_ conformation, but the other rotatable bonds were set to be flexible for the calculation. All of the protein residues were kept rigid. The cubic energy grid was centered at the substrate binding pocket and had an extension of 40 Å in each direction. A total of 256 docking runs were performed with the Lamarckian genetic algorithm.

## Abbreviations

cABC

Chondroitinase ABC;

cABC-I

Chondroitinase ABC I;

cAC

Chondroitinase AC;

CS

chondroitin sulfate;

CSase

Chondroitin sulfate lyase;

CSA

Chondroitin sulfate A;

CSC

Chondroitin sulfate C;

DS

Dermatan sulfate;

GAG

Glycosaminoglycans;

GalNac

N-acetyl-D-galactosamine;

GlcA

D-glucuronic acid;

HA

Hyaluronan;

hCSA homo-geneous CSA; hCSC homogeneous CSC;

HS

Heparan sulfate;

IdoA

L-iduronic acid;

KS

keratin sulfate;

LDH

Lumbar disc herniation;

Mw

Molecular weight;

NP

Nucleus pulposus

## Supplementary Material

Supplemental_data_Glycobiology_cwab086

## References

[ref1] Dische Z . 1947. A new specific color reaction of hexuronic acids. J Biol Chem. 167(1):189–198. 20281638

[ref2] Emsley P, Cowtan K. 2004. Coot: model-building tools for molecular graphics. *Acta Crystallogr*. D Biol Crystallogr. 60(12):2126–2132. 10.1107/S0907444904019158 15572765

[ref3] Gama CI, Tully SE, Sotogaku N, Clark PM, Rawat M, Vaidehi N, Goddard WA 3rd, Nishi A, Hsieh-Wilson LC. 2006. Sulfation patterns of glycosaminoglycans encode molecular recognition and activity. Nat Chem Biol. 2(9):467–473. 16878128 10.1038/nchembio810

[ref4] Hamai A, Hashimoto N, Mochizuki H, Kato F, Makiguchi Y, Horie K, Suzuki S. 1997. Two distinct chondroitin sulfate ABC lyases. An endoeliminase yielding tetrasaccharides and an exoeliminase preferentially acting on oligosaccharides. J Biol Chem. 272(14):9123–9130. 9083041 10.1074/jbc.272.14.9123

[ref4a] Huang W, Lunin VV, Li Y, Suzuki S, Sugiura N, Miyazono H, Cygler M. 2003. Crystal structure of Proteus vulgaris chondroitin sulfate ABC lyase I at 1.9 Å resolution. J Mol Biol. 328(3):623–634. 12706721 10.1016/s0022-2836(03)00345-0

[ref5] Kabsch W . 2010. XDS. Acta Crystallogr Sect D: Biol Crystallogr. 66(2):125–132. 20124692 10.1107/S0907444909047337 PMC2815665

[ref6] Kawaguchi Y, Sugiura N, Kimata K, Kimura M, Kakuta Y. 2013. The crystal structure of novel chondroitin lyase ODV-E66, a baculovirus envelope protein. FEBS Lett. 587(24):3943–3948. 24446551

[ref7] Linhardt RJ, Toida T. 2004. Role of Glycosaminoglycans in cellular communication. Acc Chem Res. 37(7):431–438. 15260505 10.1021/ar030138x

[ref8] Lowry OH, Rosebrough NJ, Farr AL, Randall RJ. 1951. Protein measurement with the Folin phenol reagent. J Biol Chem. 193(1):265–275. 14907713

[ref9] Mikami T, Kitagawa H. 2013. Biosynthesis and function of chondroitin sulfate. Biochim Biophys Acta - Gen Subj. 1830(10):4719–4733. 10.1016/j.bbagen.2013.06.006 23774590

[ref10] Morris GM, Huey R, Lindstrom W, Sanner FM, Belew RK, Goodsell DS, Olson AJ. 2009. Auto Dock4 and Auto Dock Tools4: Automated docking with selective receptor flexibility. J Comput Chem. 30(16):2785–2791. 19399780 10.1002/jcc.21256 PMC2760638

[ref11] Murata K, Yokoyama Y. 1985a. Enzymatic analysis with chondrosulfatases of constituent disaccharides of sulfated chondroitin sulfate and dermatan sulfate isomers by high-performance liquid chromatography. Anal Biochem. 149(1):261–268. 3935003 10.1016/0003-2697(85)90503-2

[ref12] Murata K, Yokoyama Y. 1985b. A high-performance liquid chromatography for constituent disaccharides of chondroitin sulfate and dermatan sulfate isomers. Anal Biochem. 146(2):327–335. 3927769 10.1016/0003-2697(85)90547-0

[ref13] Murata K, Yokoyama Y. 1986. Analysis of hyaluronic acid and chondroitin by high-performance liquid chromatography of the constituent disaccharide units. J Chromatogr. 374:37–44.

[ref14] Murshudov GN, Vagin AA, Dodson EJ. 1997. Refinement of macromolecular structures by the maximum-likelihood method. Acta Crystallogr D Biol Crystallogr. 53(3):240–255. 15299926 10.1107/S0907444996012255

[ref15] Olczyk K . 1993. Age-related changes in glycosaminoglycans of human intervertebral discs. Folia Histochem Cytobiol. 31(4):215–220. 8138003

[ref16] Ponnuraj K, Jedrzejas MJ. 2000. Mechanism of Hyaluronan Binding and Degradation: Structure of Streptococcus pneumoniae Hyaluronate Lyase in Complex with Hyaluronic Acid Disaccharide at 1.7 Å Resolution. J Mol Biol. 299(4):885–895. 10843845 10.1006/jmbi.2000.3817

[ref17] Prabhakar V, Capila I, Bosques CJ, Pojasek K, Sasisekharan R. 2005a. Chondroitinase ABC I from Proteus vulgaris: cloning, recombinant expression and active site identification. Biochem J. 386(1):103–112. 15691229 10.1042/BJ20041222 PMC1134771

[ref18] Prabhakar V, Raman R, Capila I, Bosques CJ, Pojasek K, Sasisekharan R. 2005b. Biochemical characterization of the chondroitinase ABC I active site. Biochem J. 390(2):395–405. 16108757 10.1042/BJ20050532 PMC1198919

[ref19] Raman R, Sasisekharan V, Sasisekharan R. 2005. Structural Insights into Biological Roles of Protein-Glycosaminoglycan Interactions. Chem Biol. 12(3):267–277. 15797210 10.1016/j.chembiol.2004.11.020

[ref20] Sárkány Z, Szeltner Z, Sárkány LP. 2001. Thiolate-Imidazolium Ion Pair Is Not an Obligatory Catalytic Entity of Cysteine Peptidases: The Active Site of Picornain 3C. Biochemistry. 40(35):10601–10606. 11524003 10.1021/bi010550p

[ref21] Schüttelkopf AW, van Aalten DMF. 2004. PRODRG: A tool for high-throughput crystallography of protein-ligand complexes. Acta Crystallogr D Biol Crystallogr. 60(8):1355–1363. 15272157 10.1107/S0907444904011679

[ref22] Sugahara K, Shigeno K, Masuda M, Fujii N, Kurosaka A, Takeda K. 1994. Structural studies on the chondroitinase ABC-resistant sulfated tetrasaccharides isolated from various chondroitin sulfate isomers. Carbohydr Res. 255:145–163. 8181004 10.1016/s0008-6215(00)90976-5

[ref23] Sugimura T, Kato F, Mimatsu K, Takenaka O, Iwata H. 1996. Experimental chemonucleolysis with chondroitinase ABC in monkeys. Spine. 21(2):161–165. 8720398 10.1097/00007632-199601150-00001

[ref24] Sugiura N, Setoyama Y, Chiba M, Kimata K, Watanabe H. 2011. Baculovirus Envelope Protein ODV-E66 Is a Novel Chondroitinase with Distinct Substrate Specificity. J Biol Chem. 286(33):29026–29034. 21715327 10.1074/jbc.M111.251157 PMC3190710

[ref25] Suzuki S, Saito H, Yamagata T, Anno K, Seno N, Kawai Y, Furuhashi T. 1968. Formation of Three Types of Disulfated Disaccharides from Chondroitin Sulfates by Chondroitinase Digestion. J Biol Chem. 243(7):1543–1550. 5647269

[ref26] Takahashi T, Kurihara H, Nakajima S, Kato T, Matsuzaka S, Sekiguchi T, Onaya M, Miyauchi S, Mixuno S, Horie K, et al. 1996. Chemonucleolytic effects of chondroitinase ABC on normal rabbit intervertebral discs. Course of action up to 10 days postinjection and minimum effective dose. Spine. 21(21):2405–2411. 8923624 10.1097/00007632-199611010-00001

[ref27] Vagin A, Teplyakov A. 2010. Molecular replacement with MOLREP. *Acta Crystallogr*. D Biol Crystallogr. 66(Pt 1):22–25. 10.1107/S0907444909042589 20057045

[ref28] Volpi N . 2007. Analytical Aspects of Pharmaceutical Grade Chondroitin Sulfates. J Pharm Sci. 96(12):3168–3180. 17630645 10.1002/jps.20997

[ref29] Watanabe I, Hikita T, Mizuno H, Sekita R, Minami A, Ishii A, Minamisawa Y, Suzuki K, Maeda H, Hidari IPJK, et al. 2015. Isolation and characterization of monoclonal antibodies specific for chondroitin sulfate E. Glycobiology. 25(9):953–962. 26036195 10.1093/glycob/cwv039

[ref30] Yamada K, Tanabe S, Ueno H, Oinuma A, Takahashi T, Miyauchi S, Shieno S, Hirose T, Miyahara K, Sato M. 2001. Investigation of the short-term effect of chemonucleolysis with chondroitinase ABC. J Vet Med Sci. 63(5):521–525. 11411497 10.1292/jvms.63.521

[ref31] Yamagata T, Saito H, Habuchi O, Suzuki S. 1968. Purification and Properties of Bacterial Chondroitinases and Chondrosulfatases. J Biol Chem. 243(7):1523–1535. 5647268

[ref32] Yoshida K, Miyauchi S, Kikuchi H, Tawada A, Tokuyasu K. 1989. Analysis of unsaturated disaccharides from glycosaminoglycuronan by highperformance liquid chromatography. Anal Biochem. 177(2):327–332. 2499215 10.1016/0003-2697(89)90061-4

[ref33] Zhang H, Neau SH. 2001. In vitro degradation of chitosan by a commercial enzyme preparation: effect of molecular weight and degree of deacetylation. Biomaterials. 22(12):1653–1658. 11374467 10.1016/s0142-9612(00)00326-4

