# Supplementary material for: Substrate specificity of Chondroitinase ABC I based on analyses of biochemical reactions and crystal structures in complex with disaccharides
Source: Glycobiology. 2021 Aug 12;31(11):1571–81. doi: 10.1093/glycob/cwab086 (PMC8684500; doi:10.1093/glycob/cwab086)
Supplement: Supplemental_data_Glycobiology_cwab086 [file supplemental_data_glycobiology_cwab086.docx]

**Supplemental data**

**Table SI. Structural data collection and refinement statistics.**

| Datasets | cABC-I | ΔDi-4S  complex | ΔDi-6S  complex | ΔDi-0S  complex |
| --- | --- | --- | --- | --- |
| PDB ID | 7EIP | 7EIQ | 7EIR | 7EIS |
| Data collection statistics |  |  |  |  |
| Beamline | AR-NW12A | AR-NW12A | BL-5A | BL-5A |
| Wavelength (Å) | 1.00000 | 1.00000 | 1.00000 | 1.00000 |
| Space group | *P*2_1_2_1_2_1_ | *P*2_1_2_1_2_1_ | *P*2_1_2_1_2_1_ | *P*2_1_2_1_2_1_ |
| Unit-cell parameters |  |  |  |  |
| *a* (Å) | 48.94 | 49.27 | 49.32 | 48.79 |
| *b* (Å) | 94.24 | 94.53 | 94.78 | 94.29 |
| *c* (Å) | 229.08 | 229.23 | 229.50 | 228.99 |
| Resolution (Å) | 50.00–1.88 | 50.00–1.80 | 50.00–1.92 | 50.00–2.50 |
| (outer shell) | (1.91–1.88) | (1.83–1.80) | (1.96–1.92) | (2.60–2.50) |
| Unique reflections | 87,389 (4,384) | 100,438 (4,953) | 83,417 (4,522) | 37,662 (4,184) |
| Redundancy | 6.5 (6.5) | 6.5 (6.1) | 6.6 (6.8) | 6.5 (6.7) |
| Completeness (%) | 100.0 (100.0) | 100.0 (100.0) | 100.0 (100.0) | 99.9 (99.9) |
| *R*_merge_ | 0.135 (0.871) | 0.101 (0.814) | 0.128 (0.846) | 0.176 (0.972) |
| Mean <*I*/σ(*I*)> | 8.8 (2.0) | 11.3 (2.0) | 10.3 (2.2) | 8.2 (2.2) |
| CC(1/2) | 0.994 (0.730) | 0.997 (0.711) | 0.996 (0.746) | 0.993 (0.835) |
| Refinement statistics |  |  |  |  |
| *R*_work_ (%) | 16.9 | 17.1 | 16.6 | 20.4 |
| *R*_free_ (%) | 21.3 | 21.4 | 21.0 | 27.8 |
| No. of molecules in ASU | 1 | 1 | 1 | 1 |
| No. of non-H atoms |  |  |  |  |
| Protein | 7,734 | 7,733 | 7,736 | 7,720 |
| Ligand | 0 | 90 | 60 | 52 |
| Solvent | 796 | 824 | 832 | 100 |
| Average B-factors (Å^2^) |  |  |  |  |
| Protein | 26.7 | 26.7 | 33.0 | 45.4 |
| Ligand | - | 34.0 | 49.5 | 63.0 |
| Solvent | 31.4 | 31.5 | 32.3 | 32.4 |
| r.m.s.d. from ideality |  |  |  |  |
| Bond length (Å) | 0.014 | 0.017 | 0.013 | 0.011 |
| Bond angles (º) | 1.798 | 2.074 | 1.901 | 1.850 |
| Ramachandran plot |  |  |  |  |
| Favored (%) | 96.0 | 96.3 | 96.3 | 92.8 |
| Allowed (%) | 3.8 | 3.4 | 3.3 | 6.7 |
| Disallowed (%) | 0.2 | 0.3 | 0.4 | 0.5 |
| MolProbity Clashscore | 2.41 | 2.65 | 2.59 | 4.42 |

**Table SII.** Mutagenesis studies of cABC-I and the role of the amino acid residues in previous reports.

| Mutant | Kinetic parameters | | Role of the amino residues | Reference |
| --- | --- | --- | --- | --- |
|  | *K*_m_ (μM) | *k*_cat_ (min^-1^) |  |  |
| No mutation | 1.2 ± 0.6 | 37000 ± 6500 | - | Prabhakar et al 2005a |
| R500A | 19.9 ± 1.0 | 410 ± 50 | Charge neutralization of the carboxy group | Prabhakar et al 2005b |
| H501A or K or R | NA | NA | Abstracts the proton from C5 | Prabhakar et al 2005b |
| Y508A | NA | NA | Donate a proton to the oxygen of the glycosidic bond | Prabhakar et al 2005a |
| Y508F | 36.4 ± 0.8 | 31 | Donate a proton to the oxygen of the glycosidic bond | Prabhakar et al 2005b |
| R560A | NA | NA | Donate a proton to the oxygen of the glycosidic bond | Prabhakar et al 2005b |
| H561A | 15.2 | 39100 | Interact with sulfate groups of chondroitin sulfate A | Prabhakar et al 2005a  Kawaguchi et al 2013 |
| E653A or D | NA | NA | Forms a hydrogen bond with His-501 and Arg-560 | Prabhakar et al 2005b |
| E653Q | 6.1 ± 0.4 | 1600 ± 300 | Forms a hydrogen bond with His-501 and Arg-560 | Prabhakar et al 2005b |
| H712A | 8.6 | 1140 | Not reported | Prabhakar et al 2005a |

The kinetic parameters were determined using CSCs derived from shark cartilage.

NA indicates no activity.


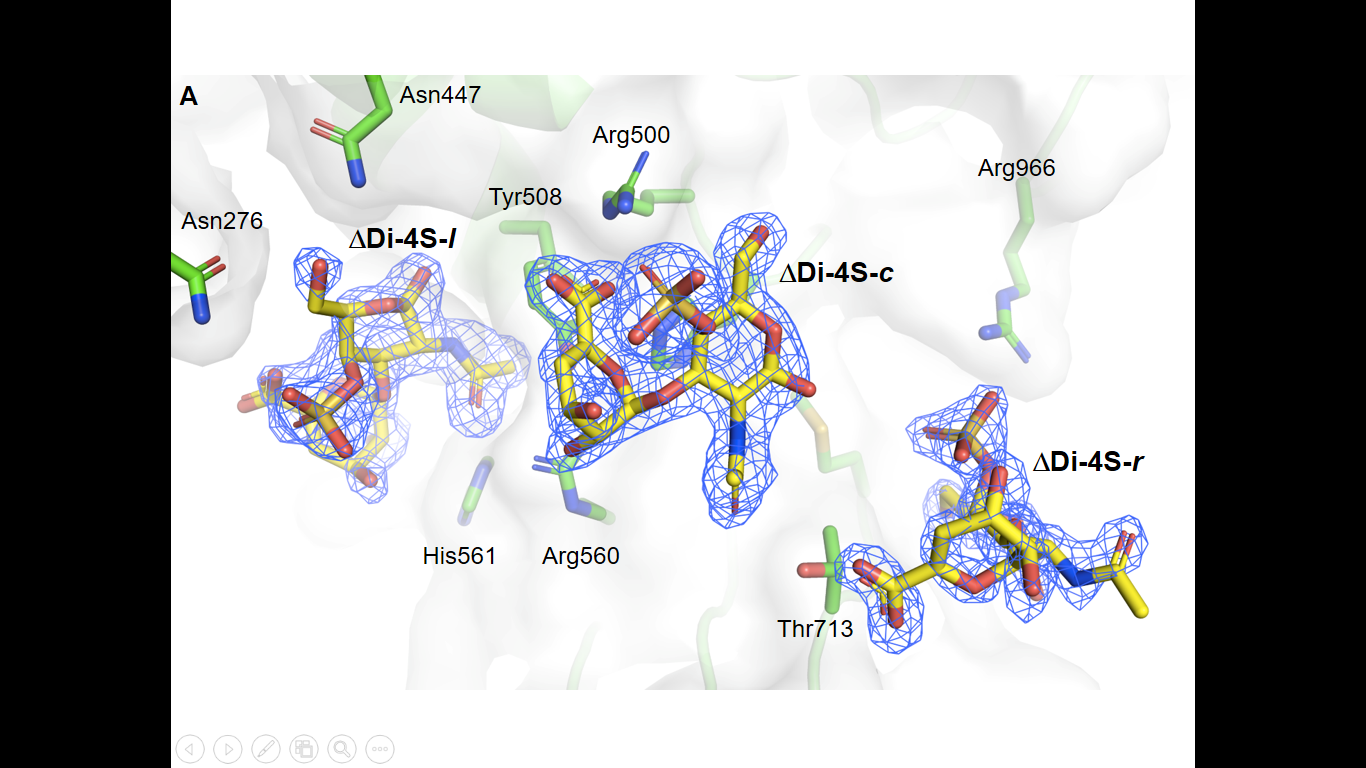


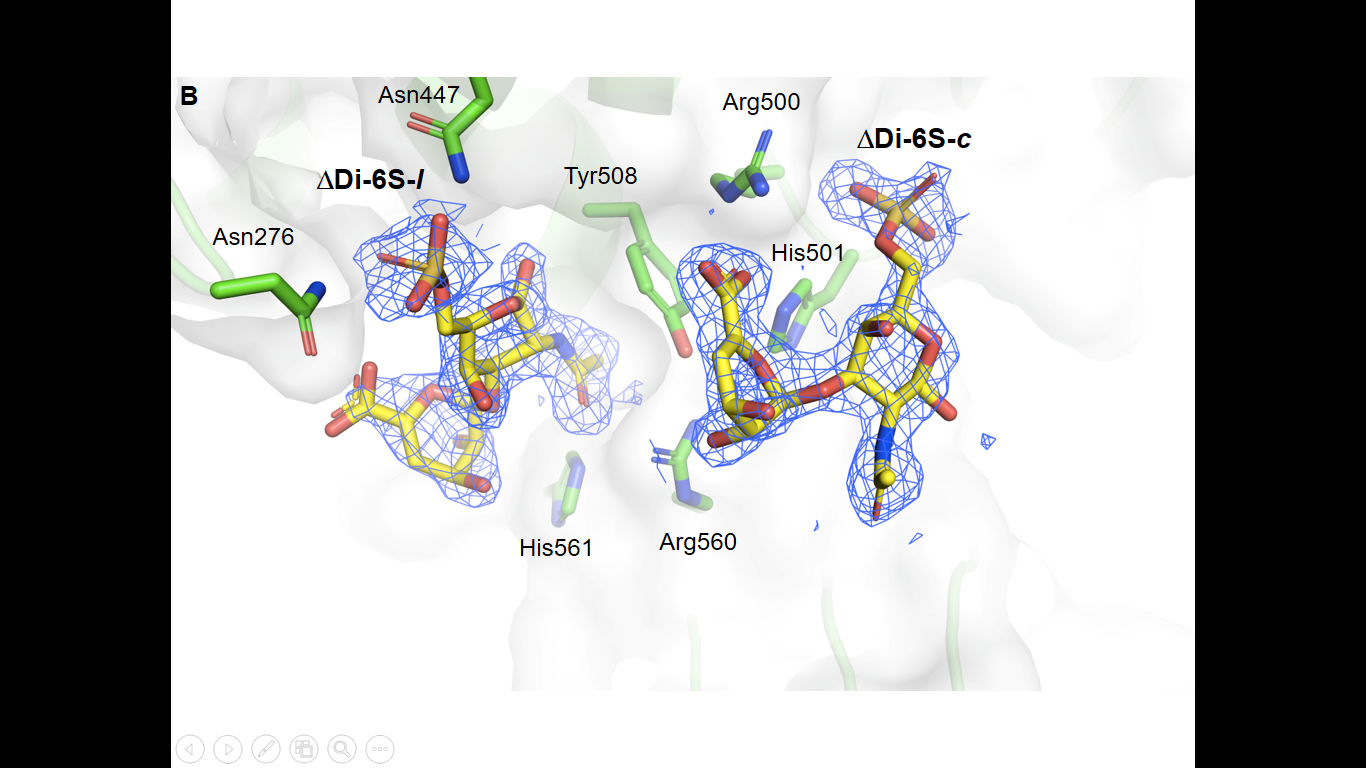


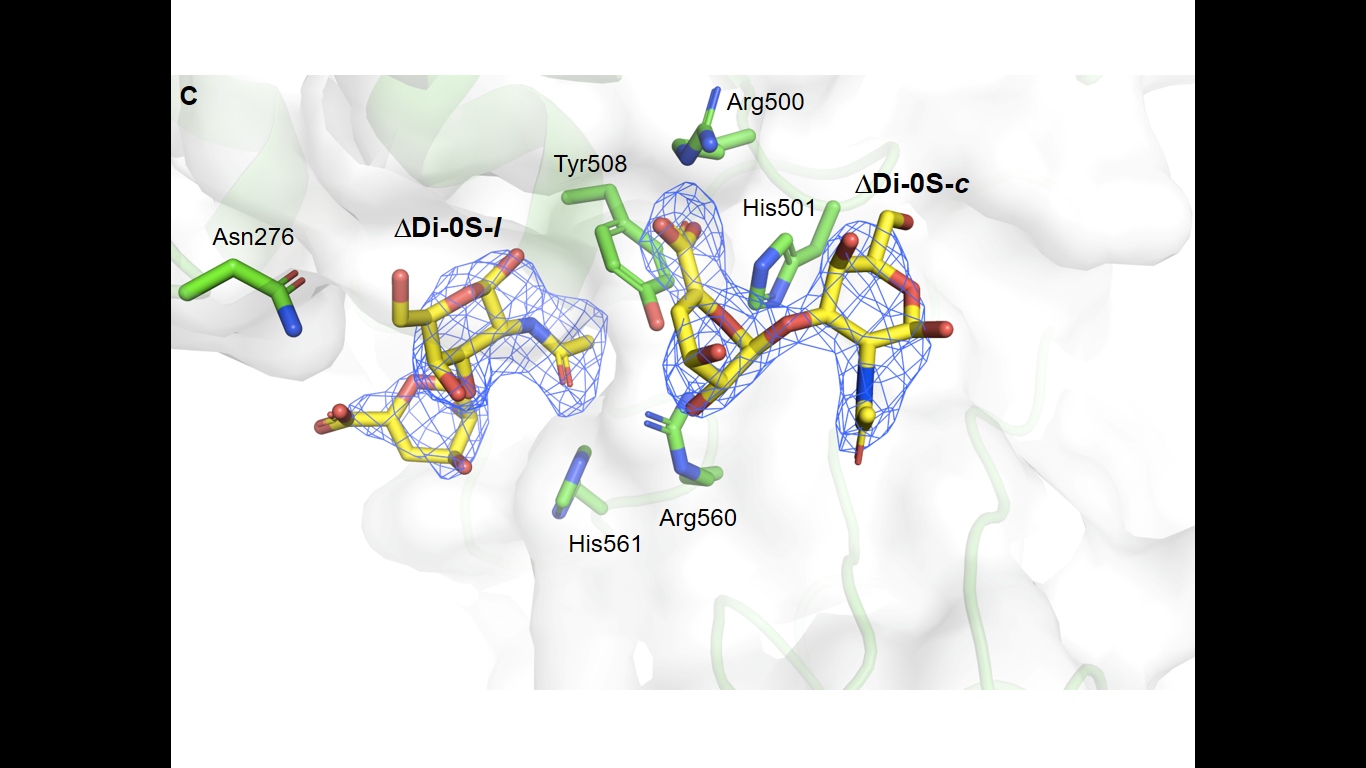


**Fig. S1. *F*_o_−*F*_c_ electron density map of the disaccharides bound to the cABC-I.**

An *F*_o_−*F*_c_ electron density map contoured at 3.0 σ was constructed for ΔDi-4S, ΔDi-6S and ΔDi-0S (A, B and C respectively).


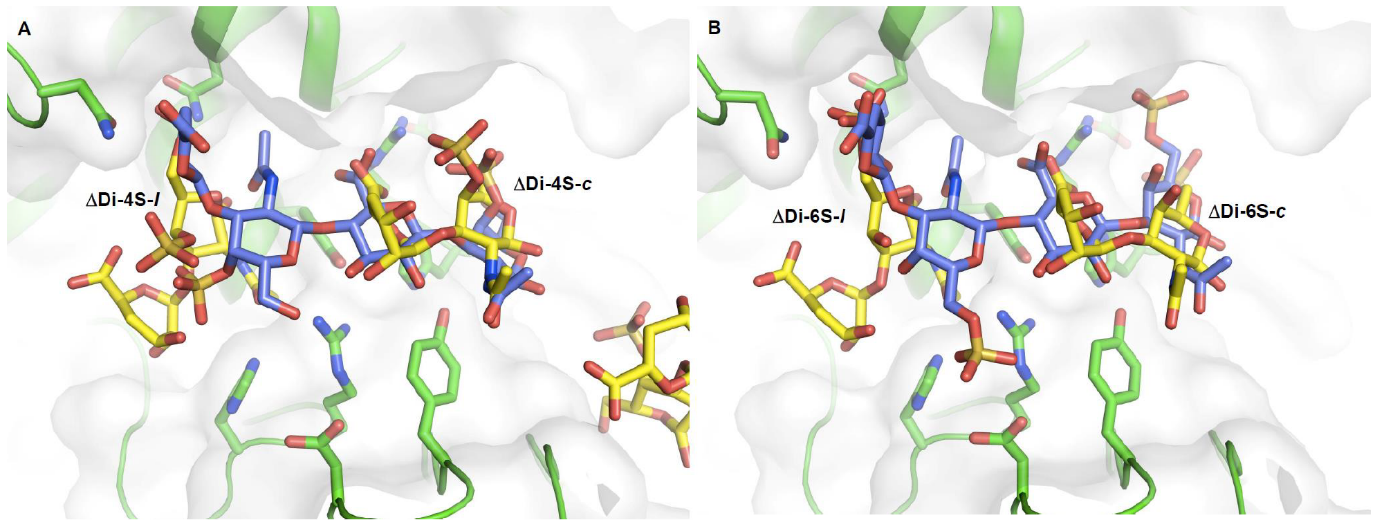


**Fig. S2. Docking analysis with CSA and CSC tetrasaccharides**

(A) Superimposition of the crystal structure of cABC-I–ΔDi-4S complex and the docking model with CSA tetrasaccharide. (B) Superimposition of the crystal structure of cABC-I–ΔDi-6S complex and the docking model with CSC tetrasaccharide. ΔDi-4S and ΔDi-6S molecules are shown as yellow sticks. Docked CSA tetrasaccharide and CSC tetrasaccharide molecules are shown as slate blue sticks.
